# WOULD YOU RATHER BE AT HOME OR IN A HOME? AN EXPLORATION AND COMPARISON OF OLD-AGE SOCIAL CONNECTEDNESS

**DOI:** 10.1093/geroni/igz038.2370

**Published:** 2019-11-08

**Authors:** Melissa Berlin

**Affiliations:** University of Michigan, Ann Arbor, Michigan, United States

## Abstract

Aging-related changes challenge older adults’ experience of social connectedness and increase their risk of perceived isolation. As seniors cope with these changes, they often face the question of whether to age in place or move to a senior living facility. While many studies explore these scenarios separately, there is little research comparing these two living arrangements. Therefore, this study examined the following questions: how do older adults experience social connectedness and perceived isolation? How does this experience vary between older adults living alone in private homes and those living in assisted living facilities? I conducted 16 qualitative interviews with middle-old and old-old women (ages 75+) who lived alone in a private home or in an assisted living facility in southeast Michigan. The themes that emerged revealed differences in three aspects of social connectedness; interactions, relationships and belonging. Community dwelling respondents’ interactions were characterized by intentionality, with minimal investment in forging new or deeper relationships and an emphasis on belonging to the world in terms of awareness, contribution, and cognitive ability. Assisted living respondents’ experiences where characterized by availability of interactions and casual relationships within the facility and an emphasis on belonging to the facility community, while positioning oneself between the status of resident and staff. Surprisingly, neither group reported a notable degree of perceived isolation. Both had adapted their social connectedness expectations to reflect their current situation. These findings have meaningful implications for older adults facing decisions about where to age, as well as for the communities that serve them.

